# Soil pH is equally important as salinity in shaping bacterial communities in saline soils under halophytic vegetation

**DOI:** 10.1038/s41598-018-22788-7

**Published:** 2018-03-14

**Authors:** Shuai Zhao, Jun-Jie Liu, Samiran Banerjee, Na Zhou, Zhen-Yong Zhao, Ke Zhang, Chang-Yan Tian

**Affiliations:** 1 State Key Laboratory of Desert and Oasis Ecology, Xinjiang Institute of Ecology and Geography, Chinese Academy of Sciences, Urumqi, 830011 China; 2 Key Laboratory of Mollisols Agroecology, Northeast Institute of Geography and Agroecology, Chinese Academy of Sciences, Harbin, 150081 China; 3 Plant-Soil Interactions, Institute for Sustainability Sciences, Agroscope, Zurich, 8046 Switzerland

## Abstract

While saline soils account for 6.5% of the total land area globally, it comprises about 70% of the area in northwestern China. Microbiota in these saline soils are particularly important because they are critical to maintaining ecosystem services. However, little is known about the microbial diversity and community composition in saline soils. To investigate the distribution patterns and edaphic determinants of bacterial communities in saline soils, we collected soil samples across the hypersaline Ebinur Lake shoreline in northwestern China and assessed soil bacterial communities using bar-coded pyrosequencing. Bacterial communities were diverse, and the dominant phyla (>5% of all sequences) across all soil samples were *Gammaproteobacteria*, *Actinobacteria*, *Firmicutes*, *Alphaproteobacteria*, *Bacteroidetes* and *Betaproteobacteria*. These dominant phyla made a significant (*P* < 0.05) contribution to community structure variations between soils. *Halomonas*, *Smithella*, *Pseudomonas and Comamonas* were the indicator taxa across the salinity gradient. Bacterial community composition showed significant (*P* < 0.05) correlations with salt content and soil pH. Indeed, bacterial phylotype richness and phylogenetic diversity were also higher in soils with middle-level salt rates, and were significantly (*P* < 0.05) correlated with salt content and soil pH. Overall, our results show that both salinity and pH are the determinants of bacterial communities in saline soils in northwest China.

## Introduction

Over 800 million hectares of land throughout the world are affected by salt^1^. Taking China as an example, northwestern China accounts for 71% of the total area of China, with 70% of the area in northwestern China comprising saline soil^2^. Saline soils are considered harsh habitats for life, but such habitats harbor active and diverse microbial communities^3–5^. It is well known that soil microorganisms are critical to maintaining ecosystem services due to their contributions to soil formation, organic matter decomposition, biogeochemical cycling and plant nutrition^6–9^. Studies in hypersaline ecosystems have characterized some enzymes and novel organisms with enhanced potential for biotechnological applications^5^. Thus, knowledge of microbial diversity in saline soil is indispensable to a comprehensive insight into the biology of extreme ecosystems.

In the past few years, reports are available on soil microbial diversity and composition from various terrestrial habitats, yielding useful insights and highlighting the importance of contemporary soil factors such as soil moisture^10^, soil trophic status^11–13^ and soil pH^14–18^. However, most of these studies focused on the microbiology of non-saline ecosystems. Only a few studies assessed the microbiology of saline environments, with the majority of them focusing on aquatic communities^19–22^ and/or sediments^23–26^, and far fewer attempting to characterize saline soils^5,27^. Thus, there is a dearth of knowledge about the structure and ecology of microbial communities in saline soils.

A recent global survey of microbial communities from natural environments found that saline soil harbors diverse bacterial communities, and that salinity was the major factor in shaping bacterial composition^28^. However, nearly half of the samples used in the study were collected from non-saline soils or different soil types. Similarly, another study reported that in soils and sediments from a hypersaline lake located in southern Texas, microbial communities were mainly driven by water content and phosphorus, rather than salt^5^. However, samples used in this study were sampled along a wide transect that spanned vegetated areas, upland areas, exposed lakebed sediments and waterlogged locations, which represent different habitats including terrestrial, intermediate and aquatic. Specifically, water influences oxygen concentrations, thus it might have stronger impacts on bacterial community structures in sediments and waterlogged locations than that of terrestrial soils. In addition, vegetated communities may result in very different microbial distribution patterns compared to upland areas. These considerations raise pertinent question about the relative influence of soil salt content as compared to other soil attributes in structuring soil microbiota in the same saline habitat. To unravel the importance of these potential drivers, our study focused on bacterial communities in the saline soil from a vegetated shoreline.

The Ebinur Lake region located in the center of an oasis, is the most representative saline ecosystem in the arid zone of China. This region has a vast area of dry lakebeds covered with halophytic vegetation that plays a key role in maintaining the regional ecological balance. It thus provides a natural laboratory for studying bacterial diversity and composition on a regional scale in a saline ecosystem. Using next-generation sequencing method, this study examined the bacterial communities in pristine soils from ten representative halophytic vegetated sites around the Ebinur Lake. Specifically, our study aimed: (i): to reveal the bacterial diversity and community composition in saline soils around the Ebinur Lake; (ii) to determine the key factors shaping bacterial distribution patterns in these soils and quantify their relative contribution to community variation. We hypothesized that salinity rather than other soil properties is more important in shaping bacterial community structure in the saline soil under halophytic vegetation.

## Results

### Soil physicochemical properties

The general physicochemical characteristics of soil samples represent the large pairwise geographical distance across 120 km (Supplementary Table S1). Soil salinity significantly (*P* < 0.05) varied from 1.77 to 8.74% (m/m) across the soil samples where the EC showed corresponding values from 4.44 to 10.83. The soil samples also differed significantly (*P* < 0.05) from each other in terms of pH (ranging from 7.94 to 8.62), organic matter (OM) (ranging from 3.32 to 9.84 g kg^−1^) or TN (ranging from 0.28 to 0.85 g kg^−1^). There was a significant (*P* < 0.05) variation in available nutrients, which ranged from 2.81 to 11.60 mg kg^−1^ for available phosphorous (AP), and from 192.11 to 453.11 mg kg^−1^ for available potassium (AK). In addition, other nutrients such as NH_4_^+^–N (ammonium nitrogen) and NO_3_^−^–N (nitrate nitrogen) significantly (*P* < 0.05) varied from 4.87 to 12.05 mg kg^−1^ and 2.98 to 9.61 mg kg^−1^, respectively.

### Distribution of taxa and phylotypes

**Figure 1:**
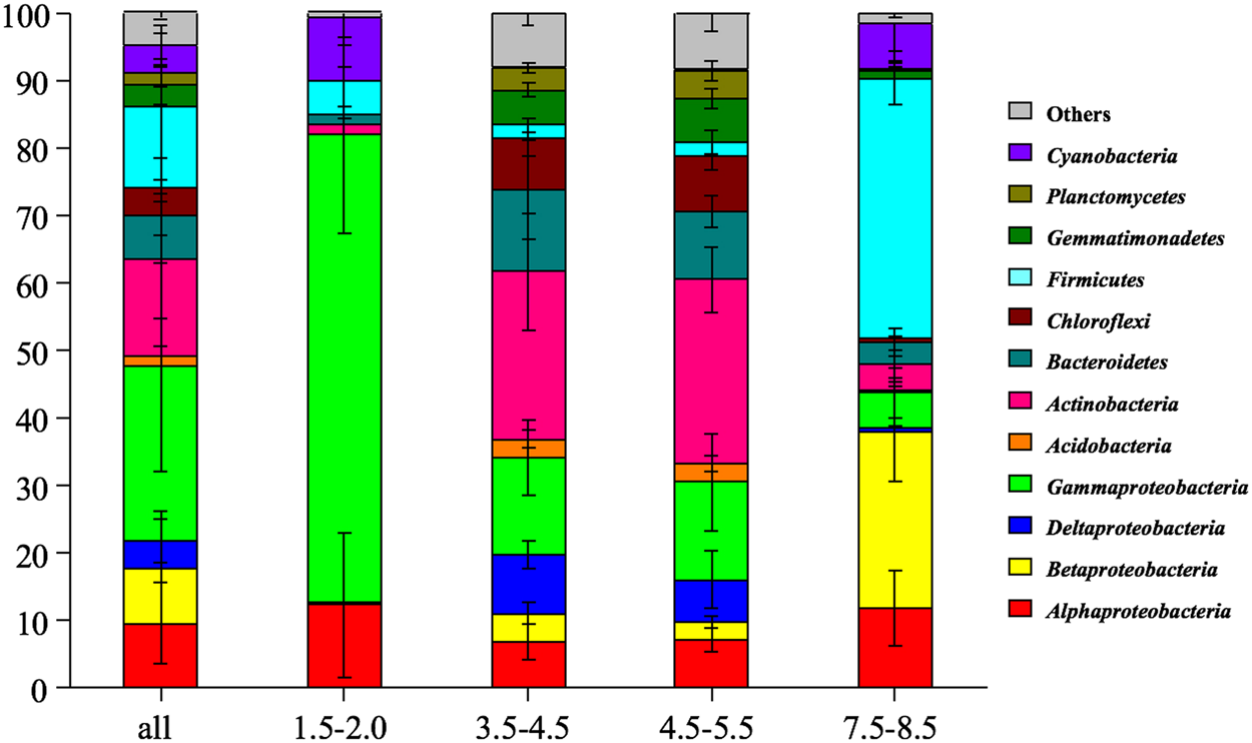
Relative abundances of the dominant bacterial groups in all soils combined and in soils separated according to salt gradients. Relative abundances are based on the proportional frequencies of those DNA sequences that could be classified. *Bars* represent standard error.

In total, 298667 quality 16S rRNA gene sequences were yielded from all 30 soil samples, and 4500–20415 sequences were obtained per sample (mean 9955). Of these sequences, when grouped at the 97% similarity threshold, there were 4389 different phylotypes over all samples, with a mean of 701 phylotypes per sample. The dominant groups (relative abundance >5%) across all soil samples were *Gammaproteobacteria* (23.55%), *Actinobacteria* (16.74%), *Firmicutes* (9.96%), *Alphaproteobacteria* (9.06%), *Bacteroidetes* (7.49%) and *Betaproteobacteria* (7.27%), which together accounted for more than 74.07% of the total bacterial sequences (Fig. 1). In addition, groups of *Acidobacteria*, *Chloroflexi*, *Cyanobacteria*, *Deltaproteobacteria*, *Gemmatimonadetes*, *Planctomycetes* and *Saccharibacteria* were present in most soils at low abundance (relative abundance >0.1%) (Fig. 1), and 20 other rarer phyla were identified (Supplementary Table S2).

The most abundant group of *Gammaproteobacteria* (r = 0.714, *P* < 0.001) were negatively correlated with salt content, while *Betaproteobacteria* (r = 0.835, *P* < 0.001) and *Firmicutes* (r = 0.813, *P* < 0.001) were significantly positively correlated with salt content (Supplementary Table S3). Interestingly, these three group (*Gammaproteobacteria*, *Betaproteobacteria* and *Firmicutes*) had the same trends towards correlating positively or negatively with soil pH. The relative abundance of *Actinobacteria* exhibited no correlation with salt content and pH, but it was significantly negatively correlated with OM (r = −0.476, *P* < 0.001), NH_4_^+^–N (r = −0.385, *P* < 0.05) and AK (r = −0.365, *P* < 0.05). Another dominant class, *Alphaproteobacteria*, had no significant correlations with any edaphic soil properties (Supplementary Table S3).

### Bacterial community structure

**Figure 2:**
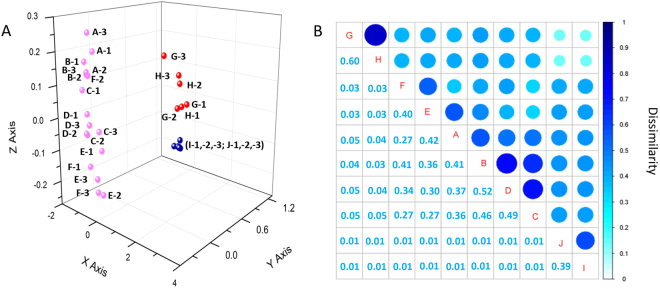
Bacterial community compositional structure in saline soils as indicated by non-metric multidimensional scaling (NMDS) plots of weighted pairwise UniFrac community distances (**A**), and the Bray-Curtis dissimilarity of bacterial community between sites (**B**). Sites have been color-coded to salt gradients.

The difference or similarity of bacterial community structures across all soil samples was illustrated by using non-metric multidimensional scaling (NMDS) plots of UniFrac community distances (Fig. 2). The NMDS plot indicated that the bacterial structure was well divided and strongly influenced by salt content (Mantel text, r = 0.712, *P* < 0.01) and soil pH (Mantel text, r = 0.602, *P* < 0.01) (Table 1). Based on the NMDS dissimilarity matrix, the bacterial communities were roughly clustered into three groups based on the NMDS dissimilarity matrix (see Supplementary Fig. S2). Group I consisted of two soils with salt content between 1.5–2.0% (Low content); Group II consisted of six soils with salt content between 3.5–5.5% (Middle content); and Group III contained two soils with salt content between 7.5–8.5% (High content). Together, the results indicated bacterial community structures were significantly different across salt content.

**Table 1:** Correlations (r) and significance (*P*) determined by Mantel tests, between the bacterial community composition and various soil environmental variables.

Variable	r	*P*
SC	**0**.**712**	0.001
pH	**0**.**602**	0.001
EC	**0**.**541**	0.001
AK	**0**.**532**	0.001
NO_3_^−^–N	**0**.**490**	0.001
OM	**0**.**423**	0.001
NH_4_^+^–N	**0**.**405**	0.001
TN	**0**.**301**	0.002
TK	0.058	0.169
AP	0.097	0.080
TP	0.087	0.072

SC, total water soluble salt content; EC, electrical conductivity; OM, organic matter; TN, soil total nitrogen; TP, soil total phosphorus; TK, soil total potassium; AP, available phosphorus; AK, available potassium; NH_4_^+^–N, ammonium nitrogen, NO_3_^−^–N, nitrate nitrogen. Values in bold indicate significant correlation (*P* < 0.05).

The SIMPER analysis was conducted to explore bacterial phyla that contributed the most to the community dissimilarity between the samples. The relative abundance of microbes and contribution of bacterial phyla between soils were analyzed separately (Table 2). Bacterial communities in the soils with a salt concentration between 3.5 and 4.5% and a salt concentration between 4.5 and 5.5% were most similar, but were different from those observed in the soils with a salt concentration of 1.5–2.0% and the soils with a salt concentration of 7.5–8.5%. The dominant phyla such as *Gammaproteobacteria*, *Actinobacteria*, *Firmicutes* and *Betaproteobacteria* made a greater contribution than other phyla to the community structure variations between soils. While the relative abundance of *Alphaproteobacteria* was greater than that of *Betaproteobacteria*, they made a smaller contribution to the community differences (Table 2, Supplementary Table S2).

**Table 2:** The respective contributions of each taxon to the variations between different soils grouped into various salt gradients.

Taxon	Respective contribution to variation (%)					
	1.5–2.0% vs 3.5–4.5%	1.5–2.0% vs 4.5–5.5%	1.5–2.0% vs 7.5–8.5%	3.5–4.5% vs 4.5–5.5%	3.5–4.5% vs 7.5–8.5%	4.5–5.5% vs 7.5–8.5%
*Alphaproteobacteria*	7.46 ± 0.08	5.72 ± 0.08	6.82 ± 0.07	1.04 ± 0.01	2.95 ± 0.02	2.74 ± 0.02
*Betaproteobacteria*	1.89 ± 0.01	1.27 ± 0.00	12.94 ± 0.04	0.91 ± 0.01	11.05 ± 0.04	11.68 ± 0.04
*Deltaproteobacteria*	4.04 ± 0.03	3.24 ± 0.02	0.23 ± 0.00	2.81 ± 0.02	3.82 ± 0.03	3.02 ± 0.02
*Gammaproteobacteria*	35.78 ± 0.10	27.55 ± 0.10	31.96 ± 0.10	3.43 ± 0.02	4.79 ± 0.03	4.69 ± 0.03
*Bacteroidetes*	5.20 ± 0.03	4.55 ± 0.01	1.20 ± 0.010	2.49 ± 0.03	4.26 ± 0.04	3.61 ± 0.01
*Firmicutes*	2.15 ± 0.02	2.18 ± 0.02	16.80 ± 0.03	0.71 ± 0.01	18.41 ± 0.02	18.27 ± 0.02
*Actinobacteria*	15.38 ± 0.04	12.73 ± 0.02	1.20 ± 0.01	3.87 ± 0.02	10.69 ± 0.04	11.69 ± 0.03
*Acidobacteria*	1.40 ± 0.01	1.38 ± 0.01	0.25 ± 0.00	0.66 ± 0.00	1.14 ± 0.01	1.12 ± 0.01
Others	2.94 ± 0.01	3.21 ± 0.01	0.57 ± 0.00	1.07 ± 0.01	2.51 ± 0.01	2.79 ± 0.01
*Cyanobacteria*	4.73 ± 0.07	4.73 ± 0.07	5.26 ± 0.06	0.05 ± 0.00	3.31 ± 0.03	3.27 ± 0.03
*Chloroflexi*	4.07 ± 0.01	3.94 ± 0.01	0.28 ± 0.00	1.39 ± 0.01	3.79 ± 0.01	3.67 ± 0.01
*Gemmatimonadetes*	2.65 ± 0.01	3.12 ± 0.01	0.54 ± 0.01	0.83 ± 0.01	2.12 ± 0.01	2.58 ± 0.01
*Planctomycetes*	1.72 ± 0.01	2.10 ± 0.01	0.13 ± 0.00	0.69 ± 0.01	1.59 ± 0.00	1.97 ± 0.01
*Verrucomicrobia*	0.78 ± 0.00	0.69 ± 0.00	0.05 ± 0.00	0.31 ± 0.00	0.73 ± 0.00	0.64 ± 0.00

Mean values are based on three replicate observations ± SD (Standard deviation).

### Indicator taxa

Taxonomic assignment of indicator species across the salt gradient has been presented in Table 3. OTU988 was the most abundant indicator taxon (relative abundance >10%) in the soils with salt concentration between 1.5 and 2.0%, and it was closely related to the genus *Halomonas*. Similarly, OTU2607 was the most abundant indicator taxon in the soils with salt concentration between 3.5 and 4.5% and it was related to the genus *Smithella*. At the next salinity level (4.5 and 5.5%), the most abundant indicator taxon was OTU3196, which was closely related to the genus *Pseudomonas*. OTU1657, closely related to the genus Comamonas, was the most abundant (relative abundance >20%) indicator taxon in soils with salt concentration between 7.5 and 8.5%.

**Table 3:** Indicator taxa identified at various soil salt concentrations. At each level, only the significant indicators were listed.

SC (%)	Indicator species^a^	Indicator value	*P*	Relative abundance (%)	Taxonomy^b^
1.5–2.0	OTU988	0.997	0.001	10.89	g_*Halomonas*
	OTU2291	0.987	0.001	8.51	g_*Halomonas*
	OTU3947	0.816	0.001	8.42	c_*Cyanobacteria*
	OTU2669	0.716	0.003	7.59	s_*Oryza meyeriana*
	OTU3888	0.705	0.004	6.33	g_*Halomonas*
	OTU2423	0.605	0.005	1.54	s_*Bacillus atrophaeus*
	OTU3764	0.601	0.034	1.48	g_*Ochrobactrum*
3.5–4.5	OTU2607	0.704	0.019	3.12	g_*Smithella*
	OTU2012	0.697	0.023	1.92	g_*Smithella*
	OTU2313	0.704	0.038	1.79	g_*Pseudomonas*
	OTU2691	0.893	0.001	1.52	f_OM1 clade
	OTU883	0.746	0.003	1.43	f_OM1 clade
4.5–5.5	OTU3196	0.714	0.037	2.46	g_*Pseudomonas*
	OTU3564	0.803	0.001	1.82	f_OM1 clade
	OTU601	0.721	0.023	1.70	f_OM1 clade
	OTU2230	0.757	0.003	1.44	f_OM1 clade
7.5–8.5	OTU1657	0.987	0.001	22.44	g_*Comamonas*
	OTU3962	0.977	0.001	18.22	g_*Lactococcus*
	OTU414	0.977	0.001	6.79	c_*Cyanobacteria*
	OTU4043	0.917	0.001	6.08	g_*Lactococcus*
	OTU3031	0.987	0.001	4.86	g_*Enterococcus*
	OTU2823	0.987	0.002	1.82	g_*Caulobacter*
	OTU1468	0.716	0.003	1.65	g_*Lactococcus*
	OTU2378	0.705	0.003	1.43	g_*Comamonas*
	OTU1123	0.690	0.024	1.10	g_*Acinetobacter*

SC, total water soluble salt content.

^a^OTUs, the relative abundance of which were less than 1%, were not listed.

^b^Taxonomic level: c, class; f, family; g, genus; s, species.

### Bacterial diversity

**Figure 3:**
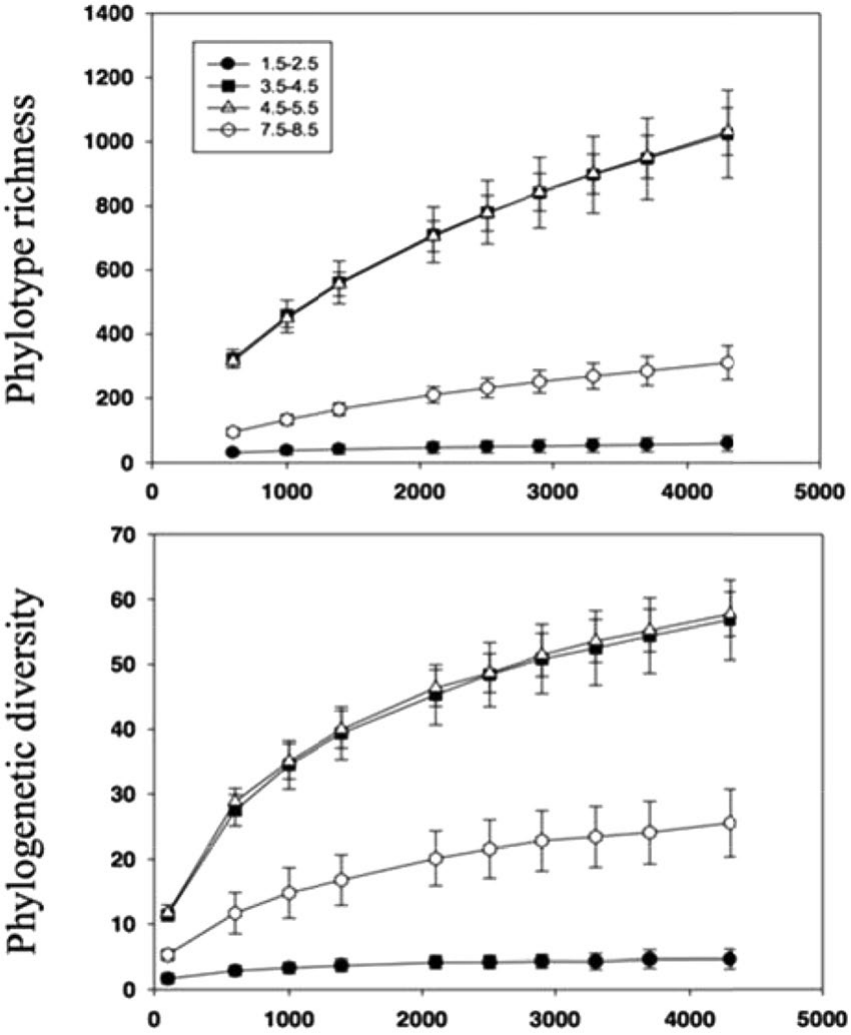
The effect of salt content on bacterial operational taxonomic unit phylotype richness and phylogenetic diversity. Diversity indices were calculated from random selections of 4500 sequences per soil sample (mean ± SE; *n* = 3).

**Figure 4:**
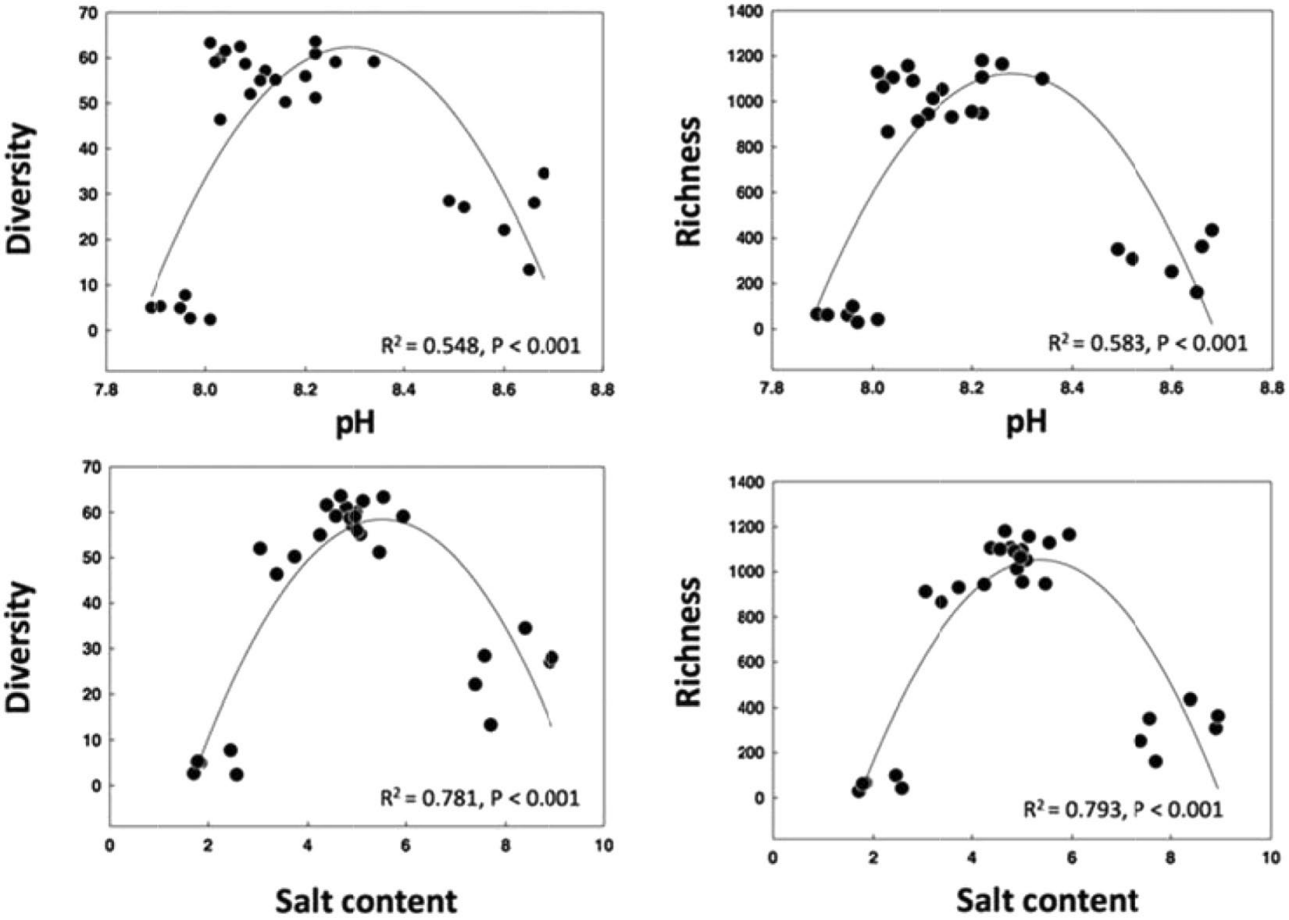
The relationship between salt content, soil pH and phylotype richness and phylogenetic diversity of bacterial operational taxonomic units. The communities were randomly sampled at the 4500 sequences level.

The diversity of the bacterial communities was highly variable in terms of both phylotype richness and phylogenetic diversity across the sample set (Figs 3 and 4). Each site contained 300–700 unique phylotypes within the 4500 randomly selected sequences per soil. Phylotype richness and phylogenetic diversity were generally higher in soils with salt concentration between 3.5 and 5.5%, while the lowest phylotype richness and phylogenetic diversity was found in soil samples with low salt content (Group I, salt concentration was between 1.5 and 2.5%) (Fig. 3). Regardless of the diversity metric employed, the bacterial diversity was best expressed by quadratic equations and it was strongly correlated with soil salt (R^2^ = 0.781, *P* < 0.001 for phylogenetic diversity; R^2^ = 0.793, *P* < 0.001 for phylotype richness) and soil pH (R^2^ = 0.548, *P* < 0.001 for phylogenetic diversity; R^2^ = 0.583, *P* < 0.001 for phylotype richness) (Fig. 4). Together, these results strongly suggest that local soil salt and pH play an important role in determining the soil bacterial composition and diversity among sites across Ebinur Lake.

### Edaphic drivers of bacterial community structure

**Figure 5:**
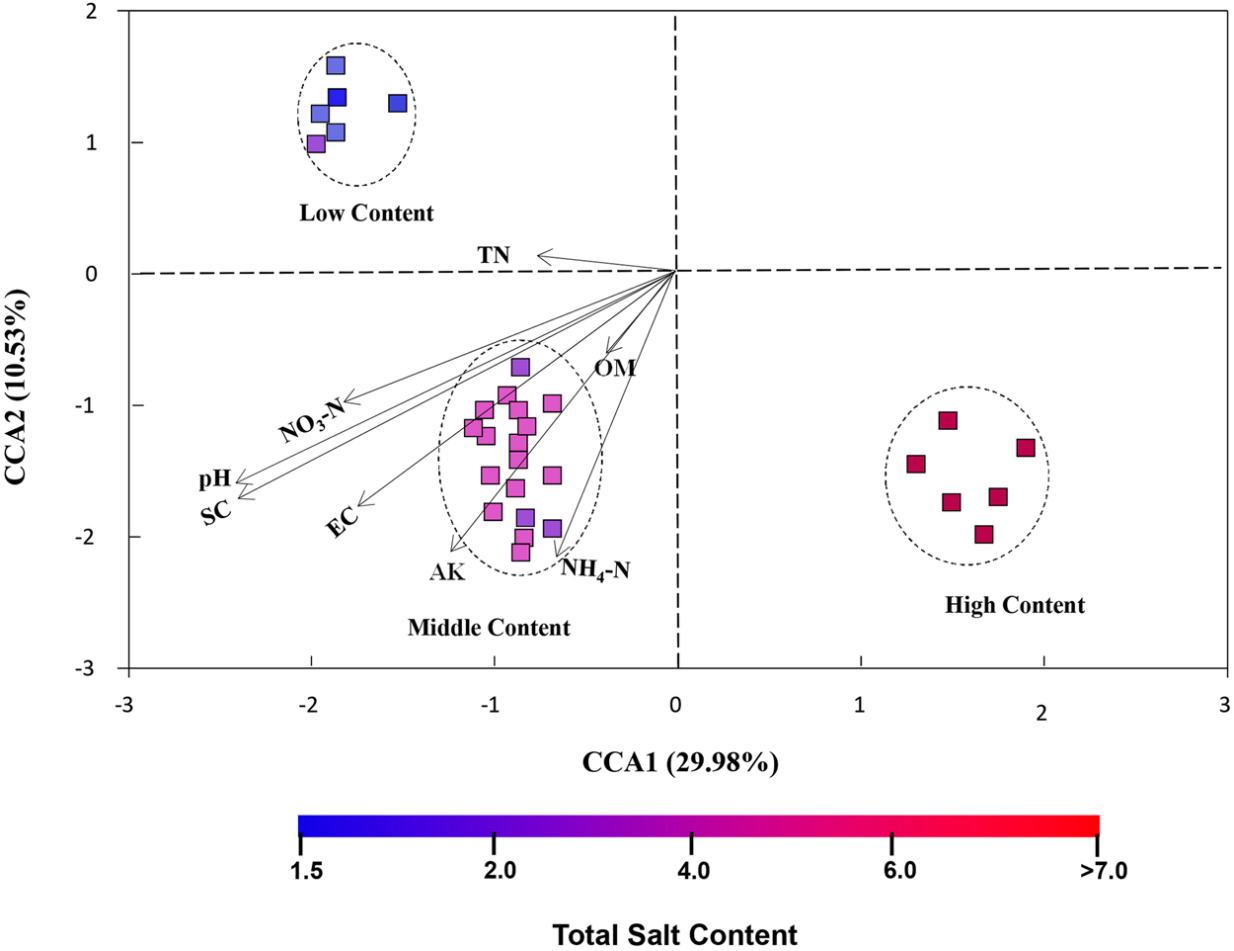
Canonical correspondence analysis (CCA) of bacterial communities and environmental variables. SC, total water-soluble salt content; EC, electrical conductivity; OM, organic matter; TN, soil total nitrogen; AK, available potassium; NH_4_^+^–N, ammonium nitrogen, NO_3_^−^–N, nitrate nitrogen.

Bacterial communities were sharply varied with salt content gradient, and the Mantel test showed there was a significant correlation between bacterial communities and salt concentration (r = 0.712, *P* < 0.001). Bacterial community composition was also significantly correlated (*P* < 0.001) with soil pH, EC, NH_4_^+^–N, AK, NO_3_^−^–N, OM and TN (Table 1). Of these variables, soil pH showed the highest correlation with the bacterial community composition (r = 0.602, *P* < 0.001). Based on the results of the Mantel test, the soil parameters significantly correlated with bacterial community structures were selected to perform canonical correspondence analysis (CCA). The CCA plots of bacterial community structure clearly exhibited that salt content and pH were the two longest vectors along CCA1 explaining 29.98% of variation of communities (Fig. 5). Thus, salt content and pH were the two important drivers controlling the community composition, which was then followed by other soil properties.

## Discussion

This study showed that bacterial communities in the saline soils under halophytic vegetation around Ebinur Lake are distinct and considerably different from those described in hypersaline sediments. For example, previous work found low G + C Gram-positive bacteria (a subgroup of *Firmicutes*), *Alphaproteobacteria* or *Deltaproteobacteria* was the predominant group in many saline sediments^4,21,23,24^, but *Gammaproteobacteria* was the dominant phylum in the saline soils in the current study. Additionally, *Acidobacteria* was major group in the hypersaline soils and sediments across La Sal del Rey lake in southern Texas^5^, but this phylum was less abundant across the saline soil around Ebinur Lake. Interestingly, the relative abundance of *Chloroflexi*, *Gemmatimonadetes* and *Planctomycetes* at sites with mid-level salt concentration was nearly 5%, which was similar to the abundances of those phyla in agricultural black soils^29^. However, the abundances of those phyla in low salt sites or high salt sites was nearly 1%, which was similar to values of those phyla in pristine soils^14,15^. These results suggest that bacterial community composition might be strongly governed by saline environmental factors around Ebinur Lake. In addition, the deep sequencing performed here was helpful for detection of bacterial taxa not described previously in saline ecosystems. These taxa generally occurred in low abundance and included members of the *Armatimonadetes*, *Fibrobacteres*, *Microgenomates* and Candidate division OP3, suggesting the enhanced sampling depth allowed the profiling of microbial communities in greater detail. It should be noted that in this study, 4500 sequences were randomly selected per sample to calculate distances between samples, lesser than studies on non-saline soils^30^. However, the coverage ranged from 89.56% (site D) to 99.82% (site I), indicating that the sequencing depth was adequate for the purpose of this study. This was also congruent with previous studies investigating bacterial community structure in saline soil (vegetated area, 1430 sequences per sample^5^) and in saline sediments (4000 sequences per sample^17^), which had similar sequencing depth yet obtained high coverage. It is possible that salinity resulted in lower bacterial abundance in these harsh environments.

While the effect of salt concentration on microbial community structure is a pertinent issue for soil ecological studies, the effect is still unclear due to contrasting evidences^5,26^. For example, significant correlation was observed between bacterial communities and salt concentrations in sediments globally^26^, whereas salt was not a major factor shaping bacterial structures in hypersaline sediments and soils^5^. Thus, there is still an active debate about the relative contribution of salt to bacterial community dissimilarity in soils. Here, we used a phylogenetic approach to compare more subtle differences among the soil samples. Consequently, we found that the bacterial community compositions, phylotype richness and phylogenetic diversity were primarily correlated with salt. The results support our hypothesis and further emphasize the notion that soil salt plays a key role in shaping the bacterial community structure in saline soils.

Indicator taxa varied among saline soils; close relatives for many OTUs could be identified, yet some remained unidentified. Some indicator species were well known, for example, the genus *Pseudomonas* containing hundreds of described species with potential for biotechnological applications. Similarly, members of the genus *Halomonas* have been isolated from saline environments (https://www.ezbiocloud.net/taxonomy)^31^. In contrast, we know little about the indicator taxon related to genera *Smithella* and *Comamonas* (https://www.ezbiocloud.net/taxonomy)^31^. Previous studies showed that indicators in fungal communities are regulated by resource type and availability^32,33^, and thus, the variation of indicator species in this study suggests that bacterial communities might also be regulated by other soil characters beside salt content.

Bacterial taxonomic richness and phylogenetic diversity both exhibited a nonlinear pattern with increasing soil salinity in the present study. Such observation was beyond our expectations because high osmotic pressure would bring adverse effect to most bacteria. Previous work found that microbial community richness and evenness decreased along increasing waterlogged and salt-rich soils and sediments, however, the highest bacterial diversity and richness observed in the mid-level salt concertation^5^. Similar negative correlations between salinity and bacterial abundances have also been reported in sediments from Pearl River to coastal South China Sea, while groups such as *Thaumarchaeota* increased with salinity^26^. In the present study, bacterial taxonomic richness and phylogenetic diversity enriched at intermediate salt rates. Moreover, the correlation between dominant phyla and salt was similar to the correlation between these phyla and pH or nutrient content. These results suggest that the saline environment is a complex system, salinity plays a critical role in structuring bacterial communities and selecting stress tolerant groups. Although bacterial communities occurring along our study transect were mainly determined by their tolerance or affinity for salt, other soil parameters such as soil pH or nutrient content might have also contributed to the bacterial community compositions in saline soils.

The importance of soil pH for bacterial community structure is widely regarded. Recent studies demonstrated pH as the driver of soil bacterial communities across North and South America^14,34,35^, in the Arctic^15^, in alkaline lake sediments across the Tibetan Plateau^17^, across British soils^36^, in Changbai Mountain soils^18^ and in the black soils of northeast China^29^. Our multiple analyses also revealed that pH had highly significant correlations with bacterial diversity and it was the main factor shaping bacterial community composition, phylotype richness and phylogenetic diversity. Typically, salt content and pH have collinearity in alkaline soils (salt mainly consisted of carbonates and pH ranges from 9.5 to 10.5). In our study, the salt primarily consisted of chlorides or/and sulphates and there was no clear relationship between salt content and pH. Taken together, these findings imply that pH is an equally important driver of bacterial community structure as salinity.

The distribution patterns of some specific bacterial phyla across pH gradient differed from their pH responses observed in previous studies. For example, the relative abundance of *Acidobacteria* and *Bacteroidetes* has often been observed to increase towards lower pH in various soils^14–16,18^, but the relative abundance of these two phyla decreased with a decreasing of pH in the current study. Furthermore, the relative abundance of *Alphaproteobacteria* was significantly correlated with soil pH in other studies^15,18^, but these trends were not observed in our study. Additionally, *Gammaproteobacteria* was positively correlated with soil pH in black soils^29^, but this phylum was negatively correlated with higher soil pH in this study. These findings indicate that the impact of soil pH on the distribution of bacterial groups in saline soils differs from other soils. However, as stated by Rousk *et al*., we still do not know whether soil pH itself is the factor directly structuring bacterial communities or if it is indirectly integrated with many environmental factors^16^. Thus, the importance of soil pH in determining bacterial communities does not completely nullify the effect of other local features in driving the individual bacterial distribution.

Strong correlations between microbial community and nutrients such as soil carbon or nitrogen contents and phosphorus contents, as well as other characters like soil EC, were found in studies across various scales. For example, soil organic carbon and TN significantly affected the relative abundance of different dominant or subdominant phyla from a wide range of ecosystems^15,37–39^. Furthermore, soil EC was a dominant factor that drove the bacterial community composition in secondary saline soils^40^. Indeed, we found that soil EC, OM, AK, TN, NH_4_^+^–N and NO_3_^−^–N had significant correlations with relative abundances of five dominant taxa. In particular, to the best of our knowledge, the present study is the first report to show a significant relationship between bacterial phyla and AK. We speculate that K^+^ ions are critical to some bacteria (e.g. *Betaproteobacteria*, *Firmicutes*) as proton replacements to cope with high external Na^+^. Interestingly, several bacterial groups such as *Deltaproteobacteria*, *Chloroflexi*, *Gemmatimonadetes* and *Planctomycetes* showed negative correlations with OM or NH_4_^+^–N, whereas *Cyanobacteria* was positively correlated with OM and TN in this study (Supplementary Table S3), and those relationships had not been previously discerned. These results suggest that nutrients that could influence the competition between species become more important for particular bacteria at local scales in salt-rich systems. We infer that the relationships between individual bacterial groups and these soil nutrient factors are likely contributing to the overall soil bacterial community structure.

Given the pivotal role of soil moisture in structuring microbial communities in various soils^10,29^, an influence of moisture on bacterial communities was also expected in these saline soils. However, there was no significant correlation between soil moisture and bacterial diversity. Since the saline soils were sampled from vegetated shoreline, we speculate that the intensity of soil moisture gradient was not strong enough to affect bacterial communities.

It is noteworthy that soil bacterial community structure may not be directly influenced by vegetation type at a landscape scale^41^, but vegetation may regulate bacterial communities by altering the soil physical environment and by modifying the quantity and quality of substrate supply (e.g. C or N source)^42,43^. In this study, although we sampled the soil from vegetation in which at least one of the three halophytic species was common, vegetation type might have indirectly affected bacterial distribution through altering the soil C and N status. Similarly, the growth and/or biomass of the vegetation might also be important factors influencing bacterial communities. Given the limited inference space of our study, it is clear that we still have much to learn about bacterial communities and their relationships with edaphic factors in these saline soils, and to determine if our results are consistent in similar saline environments. For example, influence of soil salinity might be more important at a larger scale than at local scale, and as a result the bacterial diversity might exhibit monotonic decreasing trend with salt content. Moreover, soils in sample sites I and J in our study were covered by gravel and soil depth was less than 5 cm (at 0–15 cm depth). These sites are located in a pass between two mountain peaks (Altau and Maili), which is a well-known wind gap in China. Strong wind can cause serious soil erosion affecting the diversity and abundance of soil organisms^44^. Thus, it is possible that soil erosion caused by wind might be an important factor leading to reduced diversity in these two sites (Fig. 3). Further work is necessary to explore the consistency of such observations.

Overall, we demonstrated bacterial community characteristics in the saline soils under halophytic vegetation around Ebinur Lake. We showed that *Gammaproteobacteria* and *Actinobacteria* were the dominant phyla in this saline environment. Bacterial communities were determined by both salt content and pH, and the effect of salt content was similar to that of the soil pH. Other environmental factors might also have promoted unique bacterial communities in the saline soil. To our knowledge, this is the first study to reveal bacterial phyla were significantly related to AK. This study presented an overview of bacterial communities at the local scale in saline soils, however, it is clear that we have only just scratched the surface. As sequencing technologies continue to advance, future studies will employ latest methods such as Single Molecule Real Time sequencing as well as culture-based approaches to reveal patterns of microbial communities and putative drivers in saline soils.

## Materials and Methods

### Soil sampling

Ebinur Lake (43°380–45°520 N; 79°530–85°020E) is the largest Salt Lake in Xinjiang, China. The dry salt lakebed and the surrounding region become the salt accumulation centers to form unique salt deserts where the lacustrine facies consist of abundant salt deposits. Briefly, the halophytic vegetation mainly exists in the west shoreline, and the vegetation included *Halocnemum strobilaceum*, *Haloxylon ammodendron*, *Tamarix* spp., *Kalidium foliatum*, *Halostachys caspica* and *Salicornia europaea*. Sparse patches of bulrush (*Phragmites communis*) were also present. Due to strong evaporation, an ice-like layer of salt was formed on the surface of the soil at some points with high salt content.

Soil samples were collected from north to south across a wide area around Ebinur Lake from 24 to 26 September 2015 (see Supplementary Fig. S1). Soils were collected at ten sites in total. At each site, three 100 m^2^ areas were randomly selected. At each area, soils (0–15 cm) were collected at ten sampling points using a sterile blade and mixed to obtain one composite sample. This resulted in 30 soil samples in total. Soils were sampled at similar locations below heath halophytic vegetation in which at least one of the following plant species was common: *Halocnemum strobilaceum*, *Salicornia europaea* or *Halostachys caspica*. Solid salt crusts were removed before sampling. The composited soil samples were sieved through a 2-mm mesh to remove the plant residues and stones. A portion of each soil sample was put into a 50 mL centrifuge tube, and transferred to laboratory by using ice-box. The tubes were stored at −80 °C until soil DNA extraction. The remaining soils were tested physicochemical properties.

### Soil physicochemical property determination

Soil soluble salt content was determined after drying residue; soil moisture was detected after oven drying at 105 °C for 24 hours; available phosphorus was measured by using spectrophotometer (UVmini-1240, Shimadzu, Japan); available potassium was determined by using atomic absorption spectrum analyzer (240-AA, Agilent, USA)^45^. Soil electrical conductivity (EC) was detected by using electrolytic conductivity meter (soil:water = 1:5). Soil pH was measured by using pH meter after shaking the soil/water (1:5, w/v) suspension for 30 min. Soil total carbon (TC) and total nitrogen (TN) were measured by CN Analyzer (Vario Max CN, Elementar, Germany). Mineral N was determined by using Skalar SAN plus Segmented Flow Analyzer (Skalar Analytic B.V., De Breda, the Netherlands). Ion concentrations were detected by using inductively coupled plasma mass spectrometry (ICP-MS) (ELAN 9000/DRC-e, PerkinElmer, USA).

### Barcoded pyrosequencing

Bacterial 16S rRNA gene targeted at the hypervariable V1–V3 region was amplified by PCR using a 10-nucleotide barcoded forward primer 27 F (5′-AGA GTT TGA TCC TGG CTC AG-3′) and the reverse primer 533 R (5′-TTA CCG CGG CTG CTG GCA C-3′) for high-throughput 454 GS-FLX pyrosequencing^29^. PCR reactions were performed in a 25 μL mixture containing 1 μL of each primer at 10 μ mol L^−1^, 2 μL template DNA (20 ng μL^−1^) and 22 μL Platinum PCR SuperMix (Invitrogen, Shanghai, China). The following thermal program was used for amplification: 94 °C for 4 min, followed by 26 cycles of 94 °C for 30 s, 50 °C for 45 s and 72 °C for 1 min, followed by a final extension at 72 °C for 7 min. An equal amount of the PCR product from each sample was run on a Roche FLX 454 pyrosequencing machine at Personalbio Technology Co., Ltd., Shanghai, China.

### Processing the pyrosequencing data

The raw FASTQ data were processed using QIIME Pipeline Version 1.8.0 (http://qiime.org/tutorials/tutorial.html)^46^. The paired reads were joined with FLASH (fast length adjustment of short reads) software^47^. Briefly, only sequences >200 bp in length, with an average quality score >25 and without ambiguous base calls were reserved in the subsequent analysis. The trimmed sequences were chimera-detected and removed using the Uchime algorithm^48^. The high-quality sequences were clustered through the UPARSE pipeline (http://drive5.com/uparse/) at a 97% similarity level to generate operational taxonomic units. A representative sequence from each phylotype was aligned using the Python Nearest Alignment Space Termination (PyNAST) tool^46,49^ with a relaxed neighbor-joining tree built using FastTree^50^. The taxonomy of each phylotype was determined using the Ribosomal Database Project (RDP) classifier with a confidence threshold of 0.80 (http://rdp.cme.msu.edu/)^51^. To correct for survey effort, we used a randomly selected subset of 4500 bacterial sequences per sample for calculating both α-diversity and β-diversity analyses to compare the differences and similarities between samples. All sequences have been deposited in the GenBank short-read archive (Sequence Read Archive; accession no. SRX2730198).

### Statistical analysis

The diversity indices of Faith’s index of phylogenetic diversity (Faith’s PD)^52^ and Chao 1 index^53^ were used to compare soil bacterial α-diversity. Spearman’s correlation coefficient was used to reveal the possible correlations between the abundances of different taxonomic levels of bacteria, diversity and soil characteristics, which were calculated using the IBM Statistical Product and Service Solutions (SPSS) Statistics package for Windows (version 18.0, NY, USA). Pairwise UniFrac distances calculated for the total community analyses were visualized using non-metric multidimensional scaling (NMDS) plots as implemented in PRIMER v6^54^. The Cluster analysis of the bacterial communities based on the NMDS dissimilarity matrix was applied using the ‘picante’ and ‘vegan’ packages in the R environment. Indicator species were determined by the Dufrene-Legendre indicator species analysis method to identify OTUs that are specifically associated with the different salinity regimes^33,55^. SIMFER analysis was performed to determine the respective contribution of bacterial communities to between-treatment variations. Mantel tests, SIMFER and CCA analysis were performed using the ‘vegan’ package in the R environment (R v.3.3.1)^56^.

## Electronic supplementary material

Supplementary file
