# Supplementary material for: Soil pH is equally important as salinity in shaping bacterial communities in saline soils under halophytic vegetation
Source: Sci Rep. 2018 Mar 14;8:4550. doi: 10.1038/s41598-018-22788-7 (PMC5851986; doi:10.1038/s41598-018-22788-7)
Supplement: Supplementary file 1 — Supplementary file [file 41598_2018_22788_MOESM1_ESM.pdf]

Supplementary materials

Journal name: Scientific Reports

Manuscript title: Soil pH is equally important as salinity in shaping bacterial communities in saline soils under halophytic vegetation

Authors: Shuai Zhao[1], Jun-Jie Liu[2*], Samiran Banerjee[3], Na Zhou[1], Zheng-Yong Zhao[1], Ke Zhang[1], Chang-Yan Tian[1*]

Affiliations:

[1]State Key Laboratory of Desert and Oasis Ecology, Xinjiang Institute of Ecology and Geography, Chinese Academy of Sciences, Urumqi 830011, China

[2]Key Laboratory of Mollisols Agroecology, Northeast Institute of Geography and Agroecology, Chinese Academy of Sciences, Harbin 150081, China

[3]Plant-Soil Interactions, Institute for Sustainability Sciences, Agroscope, Zurich 8046, Switzerland

Contact:

Correspondence: Jun-Jie Liu, Key Laboratory of Mollisols Agroecology, Northeast Institute of Geography and Agroecology, Chinese Academy of Sciences, Harbin, China

Tel: +86-451- 86602737

Fax: +86-451- 86603736

E-mail: liujunjie0451@163.com

Chang-Yan Tian, State Key Laboratory of Desert and Oasis Ecology, Xinjiang Institute of Ecology and Geography, Chinese Academy of Sciences, Urumqi, China

Tel: +86-991-7885301

Fax: +86-991-7885320

E-mail: tianchangyan@hotmail.com

Table S1 Soil physical and chemical properties used in this study

Site	Location N	Location E	pH	SC (%, m/m)	EC (ms/cm)	OM (g/kg)	TN (g/kg)	NO_3^--N (mg/kg)	NH_4^+-N (mg/kg)	AP (mg/kg)	AK (mg/kg)	MS (%)
SA	44'54'	82'39'	8.14±0.12c	5.34±0.52e	7.30±0.07f	5.09±0.09c	0.55±0.10bc	4.89±0.41b	5.79±0.52ab	3.51±0.75a	229.02±10.44b	11.91±0.18d
SB	44'50'	82'45'	8.12±0.11c	5.30±0.35de	7.08±0.16ef	5.60±0.41cd	0.48±0.09b	6.61±0.68c	5.87±0.41abc	9.83±0.33c	247.29±11.04b	13.90±0.20f
SC	44'49'	82'52'	8.12±0.09c	4.46±0.27c	6.30±0.17c	3.32±0.10a	0.34±0.04a	3.66±0.55a	6.62±0.82bcd	3.52±0.51a	271.31±2.56c	13.77±0.16ef
SD	44'46'	82'55'	8.21±0.14c	4.78±0.31cd	6.72±0.11d	3.92±0.33ab	0.29±0.02a	5.92±0.79c	6.49±0.83bcd	9.23±1.29c	234.88±5.76b	13.49±0.33e
SE	44'48'	83'17'	8.09±0.07bc	3.38±0.35b	5.42±0.10b	3.50±0.43a	0.28±0.08a	3.53±0.50a	7.11±0.07d	6.02±0.32b	279.15±17.00c	15.09±0.26g
SF	44'37'	83.18'	8.10±0.09bc	4.94±0.08cde	6.91±0.11de	6.80±0.07e	0.59±0.03c	4.84±0.35b	7.04±0.37cd	6.58±0.19b	229.73±6.10b	14.71±0.14g
SG	44'40'	83'11'	8.62±0.09d	8.74±0.31g	10.83±0.19h	9.84±0.47g	0.85±0.07e	9.02±0.58d	9.20±1.32e	6.45±0.44b	443.04±2.23d	10.70±0.17b
SH	44'38'	83'11'	8.58±0.08d	7.55±0.16f	9.78±0.21g	8.93±0.18f	0.69±0.02d	9.61±0.60d	12.05±0.39f	2.81±0.32a	453.11±18.95d	9.81±0.30a
SI	45'07'	82'38'	7.94±0.04a	1.77±0.07a	4.44±0.12a	4.43±0.24b	0.29±0.03a	3.64±0.46a	4.87±0.96a	11.60±0.38d	192.11±17.99a	10.88±0.22b
SJ	45'03'	82'35'	7.96±0.05ab	2.27±0.42a	4.64±0.19a	5.99±0.76d	0.57±0.04bc	2.98±0.21a	6.91±0.63bcd	6.87±0.42b	208.78±7.47a	11.35±0.23c

SC, total water soluble salt content; EC, electrical conductivity; OM, organic matter; TN, soil total nitrogen; TP, soil total phosphorus; TK, soil total potassium; AP, available phosphorus; AK, available potassium; NH_4^+–N, ammonium nitrogen, NO_3^-–N, nitrate nitrogen; MS, moisture.

Mean values are based on three replicate observations ± SD (Standard deviation)

The letters after each number represent the results of statistical analysis. The same letter indicates that no significant difference was observed at P=0.05

Table S2 Relative average abundances of phyla across all soil samples and soils grouped into various salt gradients (values represent percentage of total non-redundant sequences). Asterisk indicates sequences classified to the domain Bacteria but not to a specific phylum.

Phylum	All	1.5-2.0%	3.5-4.5%	4.5-5.5%	7.5-8.5%
Acidobacteria	1.52	0.00	2.82	2.77	0.48
Actinobacteria	14.35	1.60	25.00	27.10	3.71
Alphaproteobacteria	9.55	12.36	6.78	7.27	11.80
Armatimonadetes	0.04	0.00	0.02	0.10	0.02
Bacteria*	1.05	0.45	1.33	1.79	0.65
Bacteroidetes	6.64	1.24	11.74	10.45	3.15
Betaproteobacteria	8.27	0.17	3.92	2.76	26.20
Candidate division OP3	0.01	0.00	0.03	0.02	0.00
Chlamydiae	0.01	0.03	0.00	0.00	0.00
Chlorobi	0.07	0.00	0.12	0.10	0.04
Chloroflexi	4.13	0.00	8.14	7.88	0.49
Cyanobacteria	4.14	9.50	0.08	0.17	6.82
Deinococcus-Thermus	0.11	0.00	0.16	0.12	0.14
Deltaproteobacteria	3.78	0.06	8.09	6.50	0.47
Elusimicrobia	0.03	0.00	0.05	0.05	0.02
Euryarchaeota	0.00	0.00	0.00	0.00	0.00
Fibrobacteres	0.04	0.00	0.09	0.08	0.00
Firmicutes	11.93	5.10	1.89	2.14	38.57
Fusobacteria	0.03	0.00	0.04	0.05	0.04
Gammaproteobacteria	25.97	69.46	14.74	14.12	5.54
Gemmatimonadetes	3.17	0.00	5.43	6.19	1.06
Gracilibacteria	0.09	0.00	0.20	0.14	0.00
Hydrogenedentes	0.08	0.00	0.08	0.18	0.05
JL-ETNP-Z39	0.08	0.00	0.14	0.15	0.02
Latescibacteria	0.01	0.00	0.01	0.00	0.01
Lentisphaerae	0.01	0.00	0.03	0.00	0.02
Microgenomates	0.02	0.00	0.03	0.02	0.01
Nitrospirae	0.27	0.00	0.44	0.61	0.05
PAUC34f	0.00	0.00	0.00	0.00	0.01
Parcubacteria	0.31	0.00	0.64	0.52	0.09
Planctomycetes	1.96	0.00	3.41	4.22	0.22
SHA-109	0.02	0.00	0.03	0.03	0.01
SM2F11	0.02	0.00	0.05	0.01	0.00
Saccharibacteria	1.48	0.00	2.84	2.89	0.19
Spirochaetae	0.00	0.00	0.00	0.00	0.02
TM6	0.01	0.00	0.01	0.01	0.03
Tenericutes	0.00	0.00	0.00	0.00	0.01
Verrucomicrobia	0.75	0.00	1.50	1.42	0.06
WCHB1-60	0.06	0.00	0.12	0.12	0.00

Table S3 Pearson correlations (r) between the relative abundances of the main bacterial phyla and soil characteristics. Values in bold indicate significant correlations (*P*<0.05).

Phylum/class	EC	SC	pH	OM	NO_3^--N	NH_4^+-N	AP	AK	TN
Alphaproteobacteria	-0.027	0.016	0.090	0.092	0.056	0.232	-0.182	0.119	0.123
Betaproteobacteria	**0.739****	**0.835****	**0.881****	**0.792****	**0.833****	**0.896****	**-0.431***	**0.914****	**0.589****
Deltaproteobacteria	0.030	-0.059	-0.164	**-0.412***	-0.179	-0.258	-0.053	-0.226	-0.312
Gammaproteobacteria	**-0.711****	**-0.714****	**-0.590****	-0.254	**-0.534****	**-0.469****	**0.578****	**-0.532****	-0.333
Acidobacteria	0.131	0.009	-0.138	**-0.431***	-0.060	-0.322	-0.002	-0.276	-0.337
Actinobacteria	-0.017	-0.091	-0.214	**-0.476****	-0.234	**-0.385***	-0.115	**-0.365***	-0.321
Bacteroidetes	-0.099	-0.103	-0.191	**-0.440***	-0.224	-0.233	-0.124	-0.161	**-0.399***
Chloroflexi	-0.042	-0.081	-0.215	**-0.524****	-0.225	**-0.367***	-0.154	-0.347	**-0.391***
Firmicutes	**0.716****	**0.813****	**0.854****	**0.870****	**0.804****	**0.830****	-0.346	**0.921****	**0.759****
Gemmatimonadetes	0.069	0.001	-0.134	**-0.398***	-0.140	**-0.374***	-0.147	-0.298	-0.235
Planctomycetes	0.002	-0.078	-0.226	**-0.477****	-0.162	**-0.413***	-0.058	**-0.370***	-0.358
Cyanobacteria	0.030	0.022	0.001	**0.375***	-0.025	0.170	0.042	-0.109	**0.405***
Others	-0.034	-0.074	-0.229	**-0.480****	-0.158	**-0.366***	-0.023	-0.310	**-0.399***

SC, total water soluble salt content; EC, electrical conductivity; OM, organic matter; TN, soil total nitrogen; TP, soil total phosphorus; TK, soil total potassium; AP, available phosphorus; AK, available potassium; NH_4^+–N, ammonium nitrogen, NO_3^-–N, nitrate nitrogen.

Bold values with "*" indicates the significance at the 0.05 level.

Bold values with "**" indicates the significance at the 0.01 level.

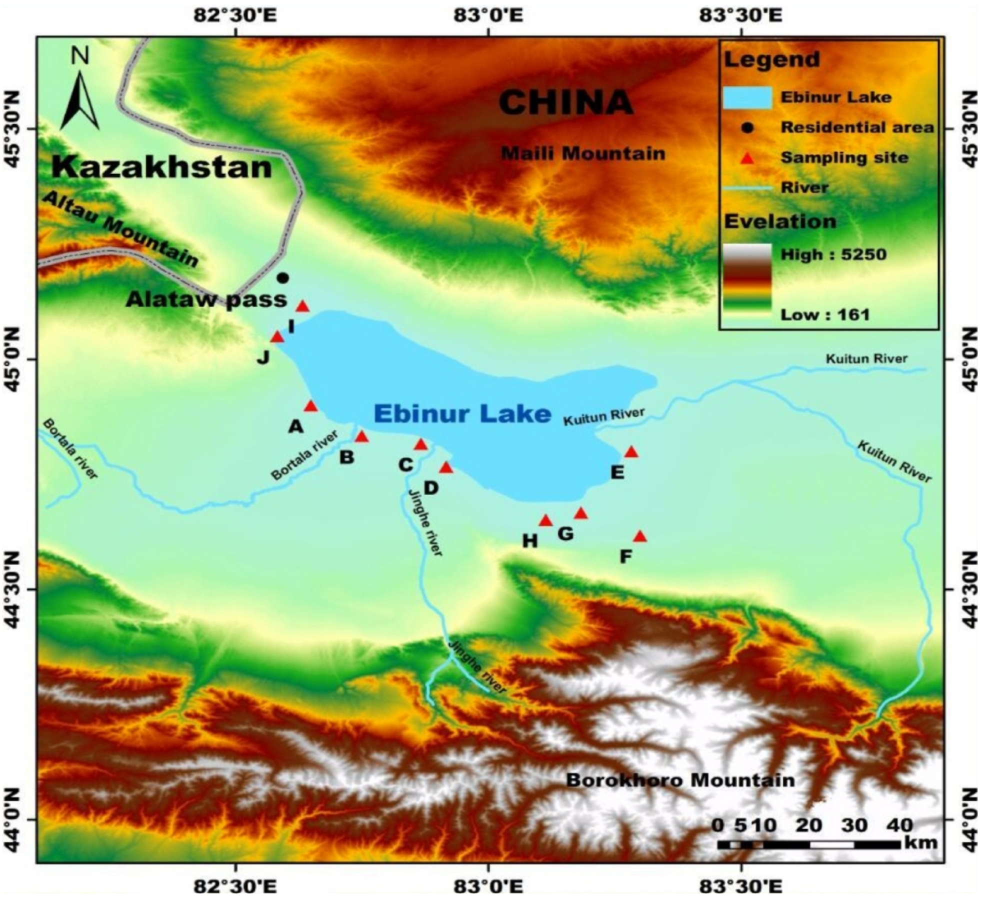

Fig. S1. Map of the sampling sites across hypersaline Ebinur Lake. ESRI's ArcMap was used to generate this figure at http://www.esri.com/en/arcgis/products/arcgis-pro/overview, and the vision of the software is 10.2.

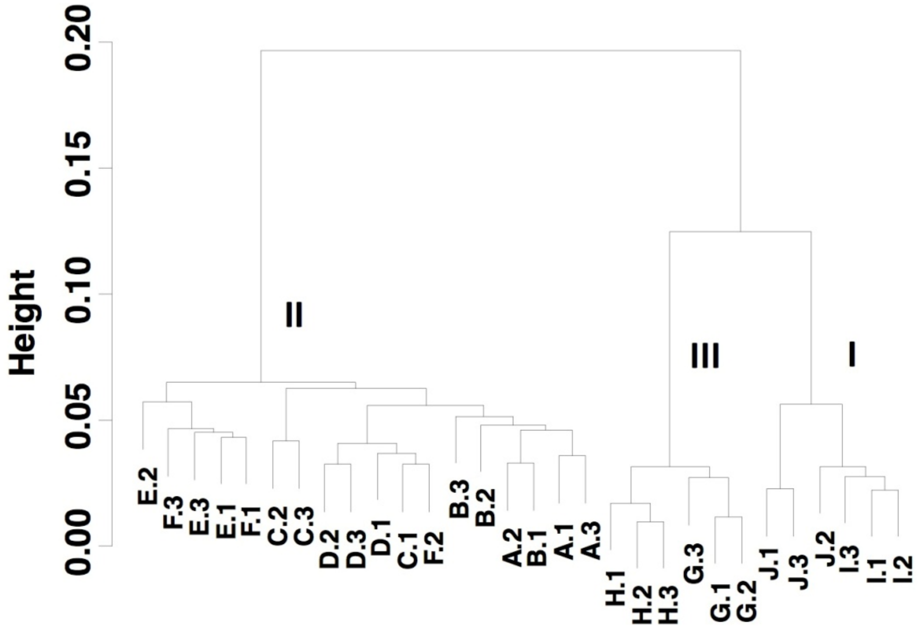

Fig. S2. Cluster analysis of bacterial communities based on NMDS (non-metric multidimensional scaling) dissimilarity matrix.
